# Historical marine footprint for Atlantic Europe, 1500–2019

**DOI:** 10.1007/s13280-023-01939-9

**Published:** 2024-01-28

**Authors:** Poul Holm, Patrick Hayes, John Nicholls

**Affiliations:** 1 Trinity Centre for Environmental Humanities, A6.002, Trinity College Dublin, 2 College Green, Dublin 2, D02 PN40 Ireland; 2 University of Victoria, British Columbia, Office B225, David Turpin Building, 3800 Finnerty Rd, Victoria, BC V8P 5C2 Canada; 3 Trinity Centre for Environmental Humanities, A6.003, Trinity College Dublin, 2 College Green, Dublin 2, D02 PN40 Ireland

**Keywords:** Consumption footprint, Fish, Food history, Marine environmental history, Marine exploitation, Seafood consumption

## Abstract

Over the last 500 years, Europe (excluding Russia) consumed over 2500 million tonnes of ocean biomass. This is based on detailed historical data that we provide for human consumption per capita which was stable from 1500 to 1899 and tripled in the twentieth century. In the last 300 years, cod and herring dominated human seafood consumption. Whaling for non-food uses peaked in the 1830s and declined as cetaceans became scarce. Seafood consumption increased rapidly after 1900, and by the late 1930s, annual marine consumption in Atlantic Europe reached 7 million tonnes of biomass, facilitated by the globalisation of whaling. Atlantic European consumption, including fishmeal for animal feed, peaked at more than 12 million tonnes annually in the 1970s, but declined thereafter. The marine footprint of Atlantic Europe was significant well before modern fisheries statistics commenced. Our findings can inform future assessments of ocean health and marine life’s importance for human society.

## Introduction

Marine environmental historians and ecologists have proposed a research agenda looking at human extractions from the sea going back centuries (Schwerdtner Manez et al. 2014; Caswell et al. 2020; Holm et al. 2022). Identifying the human footprint in the oceans drives this agenda—how much biomass have we removed, and what are the consequences for species abundance and distribution? Our study provides the first attempt to calculate Atlantic Europe's historical marine footprint based on human extraction of marine animals, including whales, between 1500 and 2019 for food and non-food uses (Online Resource 2023). We also estimate the total European marine footprint through five centuries.

No systematic assessment of the contribution of seafood to the European diet over the last few centuries is available. Guillen et al. (2019) calculated a Global Seafood Consumption Footprint for 2011, defined as the sum of nations’ consumption of biomass of domestic and imported seafood, including feed, using FAO data calibrated to live weight. The authors did not consider the historical trajectory of demand. Trentmann’s (2012) review of consumption history does not discuss fish except for a fleeting reference to the cod trade, and the standard world history of food gives a cursory reference to seafood (Kiple and Ornelas 2000). Historical estimates of national or regional consumption are rare; what estimates exist often refer to single households or institutions. Notable exceptions are Carmona and López Llosa (2009) and Grafe (2012) about Spanish fish consumption, and Morell (1989) and Neset (2010, pp. 149–179) on Swedish institutional diets. Floud et al. (2011, p. 156) estimated English food availability but repeated the figure of 24 kcal per day for fish from 1700 to 1850. Archaeological analysis has found a north–south divide in European medieval foodways with a marked Nordic preference for seafood but does not quantify overall consumption (Leggett 2022). Whaling is usually omitted in modern FAO catch data, and there are no available estimates of seafood consumption prior to published FAO statistics from 1961. Data on consumption per capita rarely include non-food. These deficiencies provide a skewed perception of the scale and trajectory of the human marine footprint, a gap which this paper fills by including both food and non-food marine extractions in a half-millennium reconstruction of the marine environmental footprint of Atlantic Europe. 

## Materials and methods

We define the human marine footprint as total human consumption in tonnes of live weight (t LW) of marine life for food and non-food purposes. The human footprint is usually calculated as consumption in hectares of equivalent land area, but this method is problematic for aquatic resources. To correct this, researchers have attempted to estimate a fishing-grounds footprint for sustainable catches (Čuček et al. 2012). Another ecological impact approach is to quantify trophic level composition of consumption. Unfortunately, patchy historical information and poor species specificity preclude such avenues, but detailed analysis may pertain to certain regions. The human marine footprint defined here is similar to the measure proposed by Guillen et al. (2019).

We selected fourteen European countries bordering the North Atlantic with significant historical records of engagement with the sea and readily available documentation. The countries explored are the modern nations of Iceland, Norway, Sweden, Denmark, Spain, Portugal, Germany, France, Belgium, Luxembourg, the Netherlands, England, Scotland, and Ireland (in short, Atlantic Europe). The data relates to boundaries of modern states, providing the available information. We present all study data online in open-access format accompanied by detailed supporting documentation, including a replication package describing methods and sources used to generate our estimates (Online Resource 2023). Figures for Ireland relate to the whole island of Ireland before 1900 and to the Republic of Ireland thereafter. Figures for England and Scotland after 1900 also relate to the whole of the UK, including Wales and Northern Ireland. Belgian figures, including the Duchy of Luxembourg, are only available after 1831 when the Southern Netherlands seceded from the United Kingdom of the Netherlands. The FAO reports Luxembourg figures separately from 2000, but we continue to combine these figures with Belgium.

**Fig. 1:**
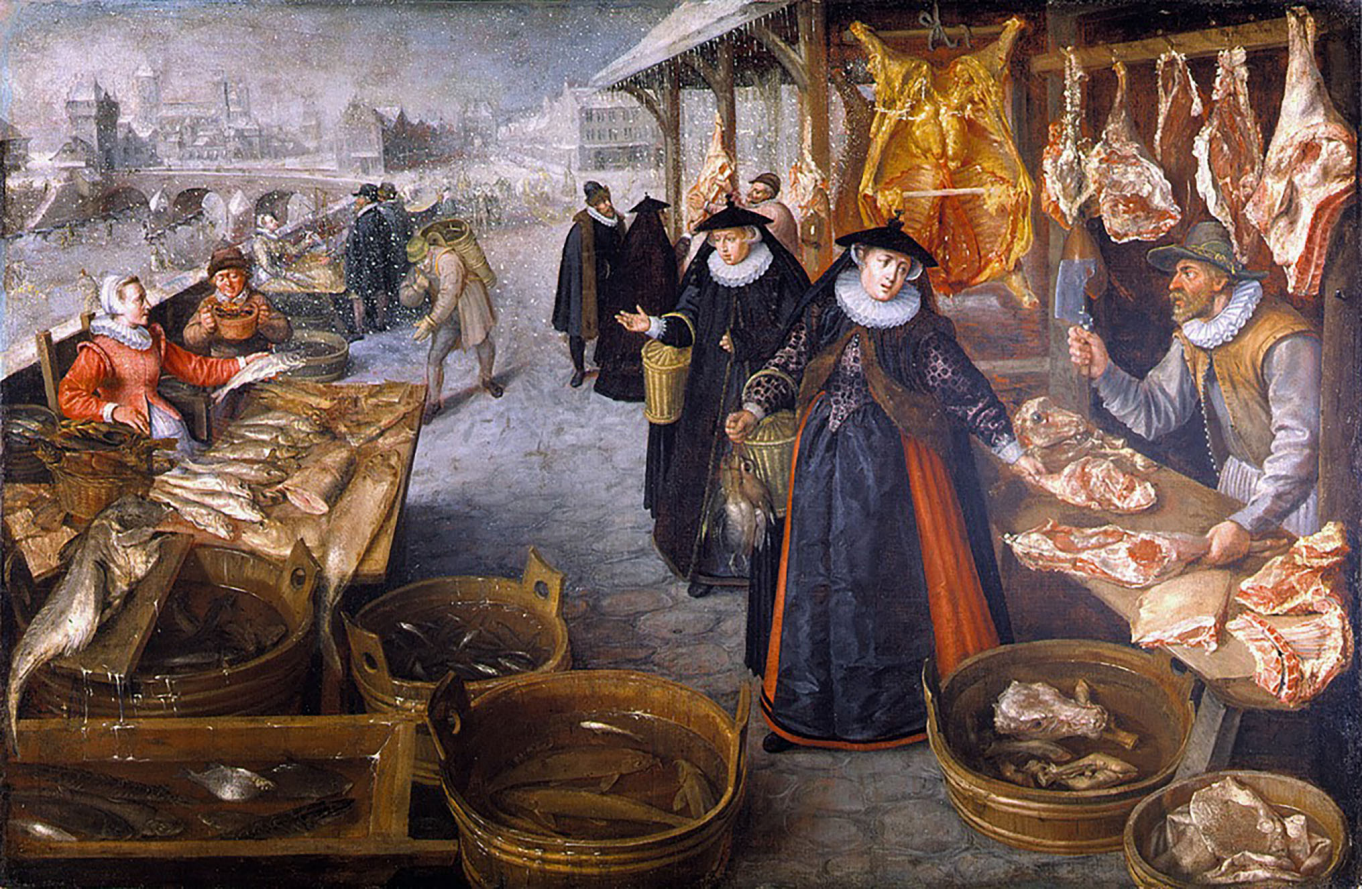
Lucas van Valckenborch, Meat and Fish Market (Winter), ca. 1595. A great variety of protein was available at the Frankfurt market. Consumer choice between seafood and meat impacted the human marine footprint

We performed a comprehensive literature survey for each country to uncover existing consumption estimates and primary sources that were used to generate new estimates. To provide consistent figures, all estimates were converted to kilograms of live weight (kg LW) using conversion factors (CF) as detailed in the Online Resource (2023). We categorise our human consumption figures into three tranches: apparent, observed and approximated estimates. Apparent consumption provides estimates based on historical records of consumption, landings, catches, imports, exports and domestic trade. Our data comprise 2382 quality-controlled records from which we estimate the supplies of seafood to populations, then divide supply by population to estimate per capita consumption (Weisell and Dop 2012, p. 157). Observed consumption considers records of the intake of individuals or defined groups, largely from representative household expenditure records. Historical data are typically only available at individual household or institutional levels. Approximated consumption figures are derived from incomplete or partial evidence and involve elements of interpretation or estimation. Distinctions are made between “national” and “partial” estimates to highlight where figures represent total seafood consumption at national levels or represent a limited number of species or a smaller section of society (Fig. 1).

**Fig. 2:**
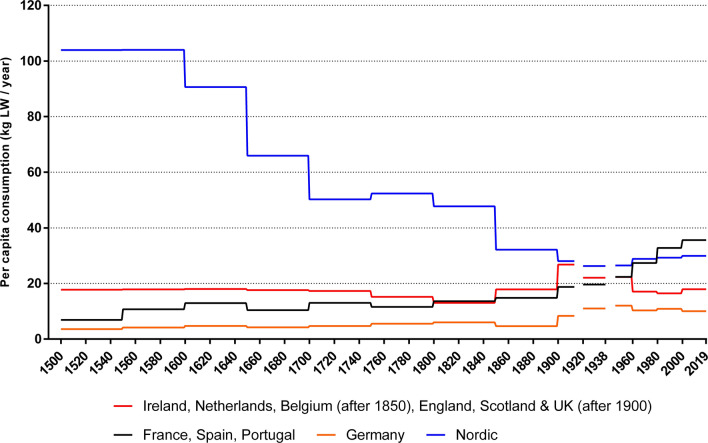
Estimated per capita seafood consumption based on 50-year averages, 1500–1899, and 20-year averages, 1900–2019, for different regions in Atlantic Europe (‘000 t LW). Figures are weighted by the total population of each nation in the region

From 1961, we deploy national apparent consumption estimates produced by the FAO by dividing the total estimated available human food supply by the population. Where usable data are absent, imputation and estimation methods have been adopted. FAOSTAT (2021) provides a full range of methods employed. Prior to 1961, we derive data from systematically reviewed published literature, complemented with our archival research. To summarise our data, we calculate average per capita consumption figures for each country over 50-year periods from 1500 to 1899. From 1900 to 2019, we use 20-year summary periods. However, we leave out the years 1914–1919 and 1939–1945 due to the disruption of the First and Second World Wars. Within the Supporting Documentation (Online Resource 2023) we score each summary estimate from 1 to 5 based on how reliable it is, with 1 being the most reliable and 5 being the least. We further specify how many data points are used to generate each summary estimate. Together these elements identify our confidence level in the robustness of each estimate. Most of our estimates fall within the high-quality levels (1 and 2), and the overall trend in consumption does not change significantly when each level is looked at in isolation. For a full exploration of the quality levels, see the Supporting Documentation (Online Resource 2023). We calculate national estimates based on low and high values in periods where there is uncertainty over consumption levels, indicating a band of deviation from the calculated averages. Our high and low estimates for 1500–1649 vary ± 25%, for 1650–1849 they vary ± 10%, while high and low estimates for 1900 onwards vary only ± 1%. From 1961 onwards, we accept FAO values as accurate with no variation. Overall per capita consumption (Fig. 2) is weighted by population, which uses historical population numbers per country from the Maddison project until 1960 (best available demographic data continually reviewed against published historical research) and from the FAO thereafter (FAO 2021a; Maddison project 2022). We estimate total seafood consumption by multiplying per capita figures by the population estimates.

**Table 1:**
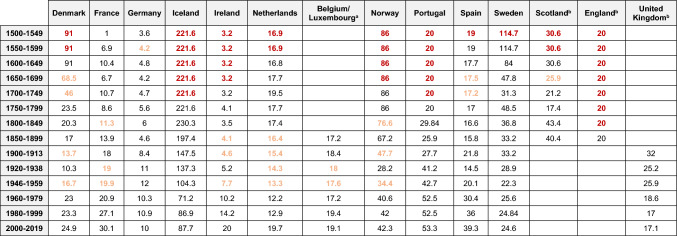
Estimated per capita seafood consumption by European Atlantic nations, 1500–2019 (average kg LW per capita). Values are colour coded to indicate reliability

Values in black are solid data points

Values in red are extrapolated backwards from the first available solid data point

Values in orange are gaps that have been filled using interpolated step values between two solid data points

For full details on all these estimates see Online Resource (2023)

^a^Between 1500 and 1831 Belgium was a part of the Netherlands

^b^Values for England and Scotland are reported jointly under the UK, but separately prior to 1900

**Fig. 3:**
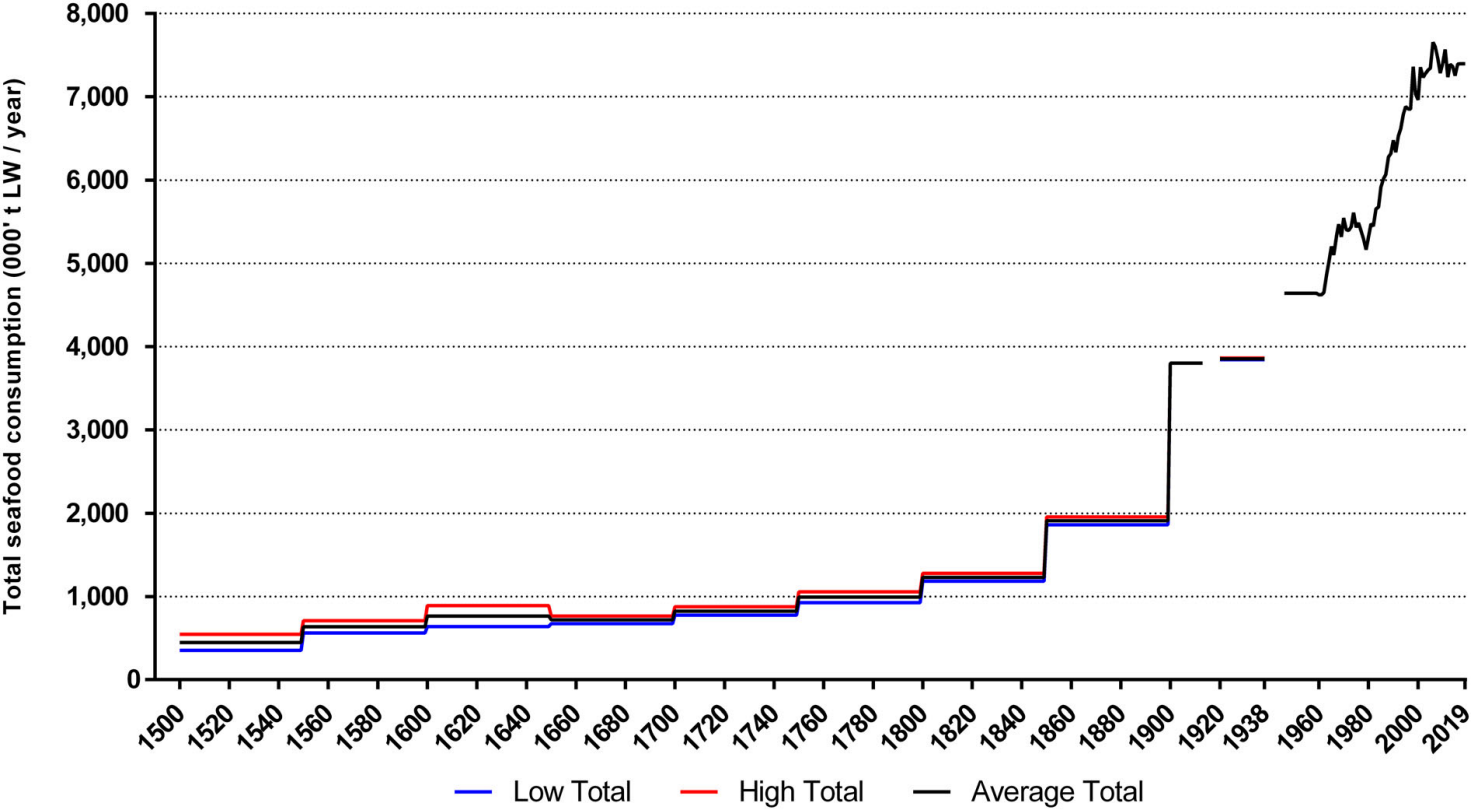
Estimated total seafood and non-food consumption 1500–2019 (‘000 t LW/year). Low and high estimates are provided until 1900

We fill gaps in data using interpolated step values between the two nearest solid data points. These values are represented in amber in Table 1. If we cannot fill such a gap, we use the first available solid estimate to extrapolate backwards in time. These values, represented in red, are only shown to produce rough estimates of total consumption (Fig. 3) and are omitted in our final per capita calculations (Fig. 2). Our data from 1750 onwards includes no extrapolated (red) values, except England, which only has solid values from 1850. More details on our data and methods can be found in the Online Resource (2023).

Since 1970, the rapid expansion of the global fish trade makes any assessment of country-specific marine footprints exceedingly difficult. We therefore refrain from making nation-specific estimates and given figures for the European global footprint only. Pre-1950, we have assumed that global marine products, apart from the well-documented whale oil trade, did not play a significant role and assumed that European catches were predominantly consumed in Europe.

Non-food uses of marine resources mainly concern the reduction of whales to train oil for lighting and the reduction of fish to meal and oil. Seals, porpoises, and occasionally whales, figured on elite menus in the medieval period, but were essentially considered non-palatable by the seventeenth century (Barlösius 2000).

Whaling data are obtained from existing studies, established databases and archival research. The IWC database provides detailed whale catch data extracted from national government statistics and spans the twentieth century (IWC Database 2022). Data include details of specific catch regions and grounds and catch breakdowns per species. eighteenth and nineteenth century data have been drawn from open-access databases of logbooks, archival documents and reports (du Pasquier 1990; BNAW 2020; Jones et al. 2020; Vieira 2023). We combined these sources to estimate the number and biomass of captured whales in the North Atlantic and global waters.

Through centuries, farmers used seaweed and shellfish to add nutrients and lime to soil, and fish were used as animal feed (Illera-Vives et al. 2020). However, we have not attempted to calculate the past consumption of aquatic plants or fish meal before the nineteenth century. The use of fish for non-food uses increased in the mid-nineteenth century with the development of the Norwegian Fish-Guano industry (Solhaug 1976). The subsequent invention of the continuous production platform in the USA during World War I facilitated the large-scale reduction of fish to fish meal and oil. The technology was transferred to Norway, which became the European industry leader. In the 1950s, Denmark and the UK developed their own industries, and the Danish industry quickly rivalled Norwegian production (Fasting 1954; Cutting 1955; Holm et al. 1998; Engelhard et al. 2014). Products were initially used as feed for pigs, poultry, and mink, and later as an essential part of the aquaculture sector. In recent years declining marine resources have caused the Scandinavian industry to scale back, while Spain has seen some growth.

FAO production statistics for fishmeal and oil are available from 1961 (FAO 2021b). We estimate Western European landings for non-food purposes for the years 1920 and 1938 based on Norwegian, Danish and UK industry figures. Production was based on offal and waste but increasingly depended on direct supplies of herring, sand eel and other small pelagics, including some gadids (Cutting 1955). Conversion ratios to live weight are elusive as they depend on fish species and production methods. Norwegian production statistics before 1953 show a ratio of 5.4 (calculated from Fasting 1954, pp. 218–219). Modern ratios vary considerably from 2.7:1 to 5.11:1 (Jackson 2009; Avadí et al. 2014), and from 1961 we have used 4.5:1.

To calculate the contribution of seafood to total human nourishment, we use FAO statistics from 1961 onwards. For premodern times, we assume an average consumption across adults and children of 2000 kcal per day (calculated from Floud et al. 2011, pp. 56, 156). The edible weight of fish is 50% of live weight (Barker 1968, p. 121), and 1 g of seafood provides 1 kcal, calculated based on FoodData Central (2023).

In 1961, according to FAO statistics, 60% of European seafood was consumed in Atlantic Europe and 40% in other European countries, excluding the Soviet Union. The resources will have come from the wider North Atlantic, including the Arctic Sea, the Baltic, the Black Sea and the Mediterranean. In the absence of comparable data, we assume the ratio was the same in previous times. We do not account for whaling/sealing and fishmeal production conducted by non-Atlantic countries. The total figures for all-European consumption post-1950 are therefore underestimated.

## Results

### Seafood consumption per capita

Table 1 reveals geographical and temporal differences in seafood consumption per capita through five centuries. In the early modern period, Iceland was extremely fish-reliant and remains so, albeit at a considerably reduced level. Other Nordic countries also had very high consumption levels in the early modern period, but Denmark moved away from seafood in the seventeenth century, and to a lesser degree, Sweden and Norway in the nineteenth century. At the other end of the spectrum, Germany and Ireland had very low seafood consumption. German consumption per capita remains low, while Ireland dramatically increased fish intake during its Celtic Tiger prosperity decade of the 1990s. English cities like London and Manchester experienced remarkable increases of seafood consumption in the mid-nineteenth century. Unfortunately, we lack data for national figures (Mayhew 1851; Scola 1992). A similar trend was seen nationally in Scotland, the Netherlands, Belgium and France by the turn of the century. After 1900, the importance of seafood increased in Portugal, and Spain to a lesser degree. Overall, however, consumption levels converged across all nations in the twentieth century. When data are considered by larger regions (Fig. 2), the decline of consumption per capita in the Nordic region and the increases in the Southwest Atlantic (France, Spain, and Portugal) are apparent.

In 1961, seafood contributed 81% of the total intake of animal protein in Portugal, while Spain (53%), Iceland (43%) and Norway (37%) were also fish-reliant. In Germany and Ireland seafood was only 10–11% of animal protein. In the other countries, seafood was 20–30% of animal protein. Due to poor historical data for animal protein, extending this analysis further back in time is impossible.

### Total consumption of seafood

Having established country-specific per capita consumption figures, we calculate overall seafood consumption in Atlantic Europe by multiplying by historical population figures (Fig. 3, Table 2). Humans in Atlantic Europe consumed about 0.45 million t of marine life annually by 1500, increasing to an annual consumption of 0.76 million t a century later. Extractions increased slowly between 1600 and 1749, albeit with a decline in the period 1650–1699. Removals increased rapidly after 1750, more than doubling to 1.9 million t by the latter half of the nineteenth century and doubling again to 3.8 million t after 1900. Levels were static in the interwar period but rose rapidly in the 1950s to a yearly average of 4.6 million t. Consumption annually exceeded 7 million t in the first decades of the twenty-first century.

**Table 2:**
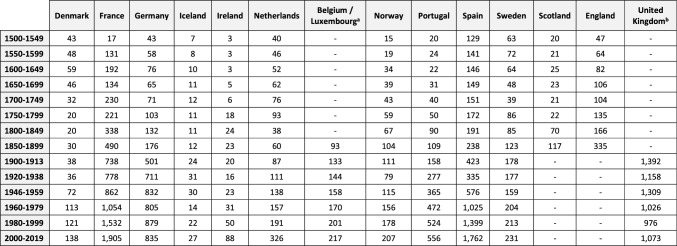
Estimates of total seafood consumption based on 50-year averages, 1500–1899, and 20-year averages, 1900–2019, for countries in Atlantic Europe (‘000 t LW per year)

^a^Between 1500 and 1831 Belgium was a part of the Netherlands

^b^Values for England and Scotland are reported jointly under the UK, but separately prior to 1900

Between 1500 and 1800, marine extractions and human population numbers largely followed the same trajectory (Pearson’s *r*^2^ = 0.91, *P* = 0.008). The nineteenth century saw a significant step change in total extractions, increasing by 52% in the first half of the century compared to the preceding half-century, and by a staggering 101% in the second half. Growth was only 25% in the first half of the twentieth century, then 57% in the second. Since 1998, the total consumption trend has become decoupled from demographic growth as seafood consumption per capita has declined.

Table 2 shows estimates of total marine consumption by nations through five centuries. Measured by overall consumption through the twentieth century, the most important seafood nations were the United Kingdom and France, accounting for 46% of total consumption. Germany and Spain constituted an additional 31%. Despite a consistently very low per capita intake, Germany was a major consumer due to its large population.

### Species-specific consumption

Seafood preferences reflect various factors, such as species abundance, availability, price, and taste. We have data going back centuries for two major commercial fish species: herring and cod (Table 3). Apparent consumption of these species was 4.8 kg per capita by the end of the Middle Ages (based on a median calculated for the decade of the 1520s). Consumption had almost doubled by the late eighteenth century. Unfortunately, we do not have landings figures for the nineteenth century, but in 1903, the first year of ICES statistics, per capita consumption was at 11.0 kg and by 1961, apparent consumption of cod and herring reached a peak of 13.7 kg per person per year (Holm et al. 2021). These average figures vary between countries. Notably, apart from the seventeenth century, landings surpassed demographic growth and enabled increased apparent per capita consumption of the two species.

**Table 3:** Consumption of cod and herring per capita, 1500–2000

Periods	Cod and herring median landings, t LW	Cod and herring kg LW p/c per annum	Cod and herring % of total seafood
1500–1549	211 100	4.8	48.2
1550–1599	322 200	6.4	51.6
1600–1649	410 100	7.5	54.3
1650–1699	350 700	5.9	49.5
1700–1749	426 400	6.9	51.3
1750–1799	649 100	8.3	66.2
1900	2 181 000	11.0	59.4
1961	3 684 300	13.7	82.6
2000	2 398 800	7.6	35.2

Herring and cod accounted for about half of all consumption between 1550 and 1700, gaining increasing importance from 1800 and dominating human seafood diets by the mid-twentieth century to the practical exclusion of other species. By 2000, Atlantic cod and herring landings declined to levels seen a 100 years earlier and contributed little more than a third of European consumer demand for seafood.

### Non-Food

**Fig. 4:**
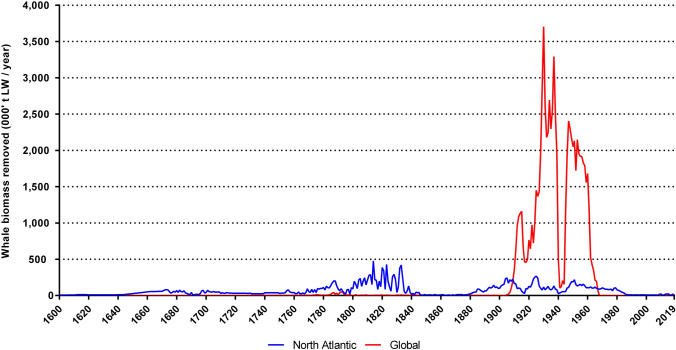
Estimated biomass harvested by European whaling in the North Atlantic and globally, 1600–2019 (‘000 t LW per year). No data are available for 1931. Global European whaling (initiated from Europe, but outside European waters) vastly surpassed North Atlantic whaling in the twentieth century

**Fig. 5:**
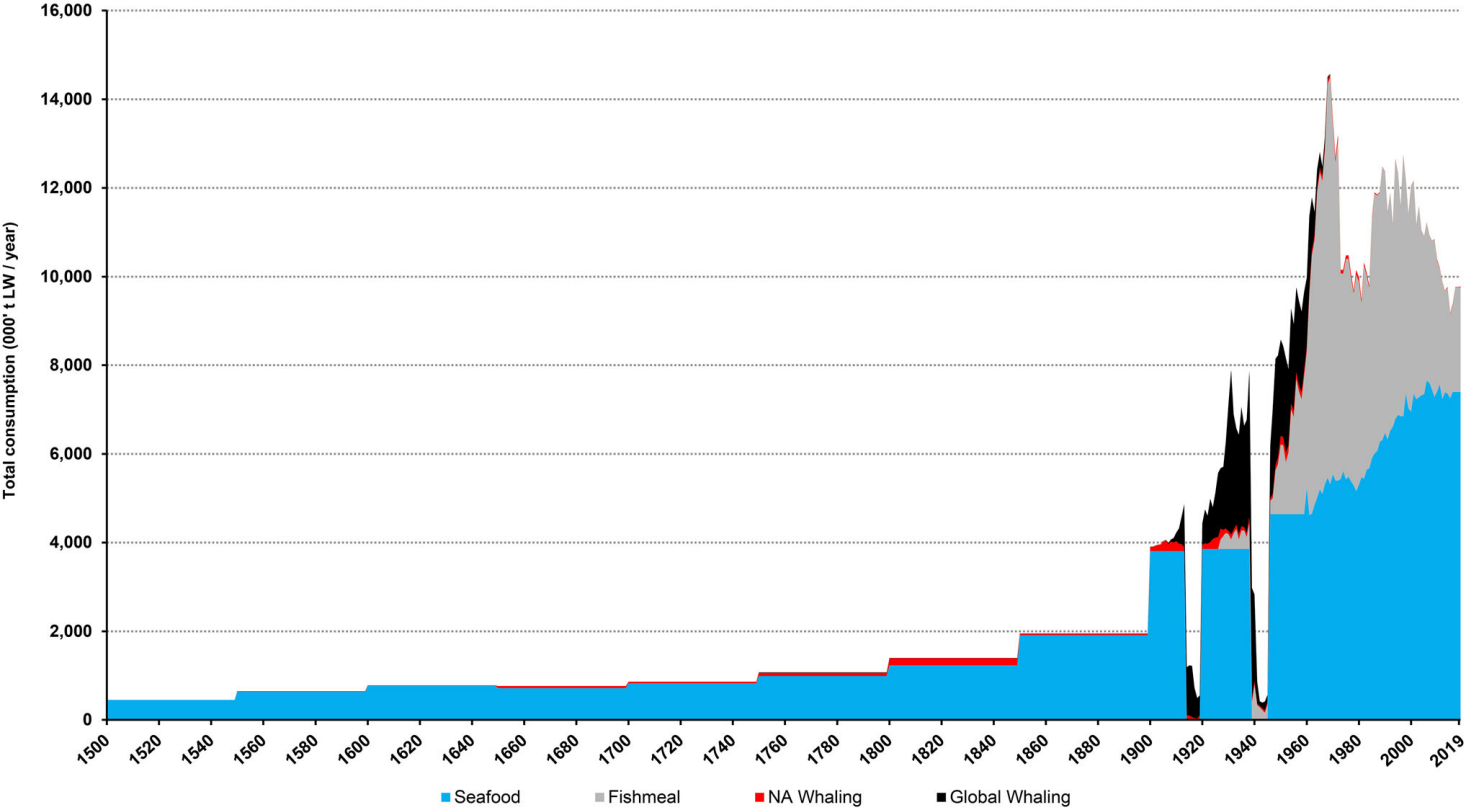
Estimated total human food and non-food marine consumption 1500 to 2019 (‘000 t LW per year). Global Whaling includes all whale biomass consumed in Europe, originating from anywhere in the world. Seafood values during the two world war periods are not shown as reliable data is not available in these years (1914–1918, 1939–1945)

Total non-food consumption is calculated based on the total biomass of whales removed from 1600 to 2019 (Fig. 4) and the production figures for fishmeal from 1927 to 2019 (Fig. 5). European whaling peaked in the 1830s based on North Atlantic catches, and again in the 1930s based on global catches, predominantly in the Southern Ocean.

We have not attempted to calculate non-food uses of fish prior to the twentieth century. Total production in 1927 amounted to 39 911 t fishmeal derived from 200 000–250 000 t live weight herring. By 1938, production had more than doubled to 88 322 t meal. The industry was at its highest levels in the 1960s and 1970s, reaching its peak in 1968 (Fig. 5); during this period, removals of fish for non-food surpassed those destined for direct human consumption. North Atlantic extractions were similar to the 1750 to 1850 period, while whaling by North Atlantic countries in other oceans accelerated massively in the twentieth century, peaking at over 3.8 million tonnes in 1930.

### Total consumption

**Fig. 6:**
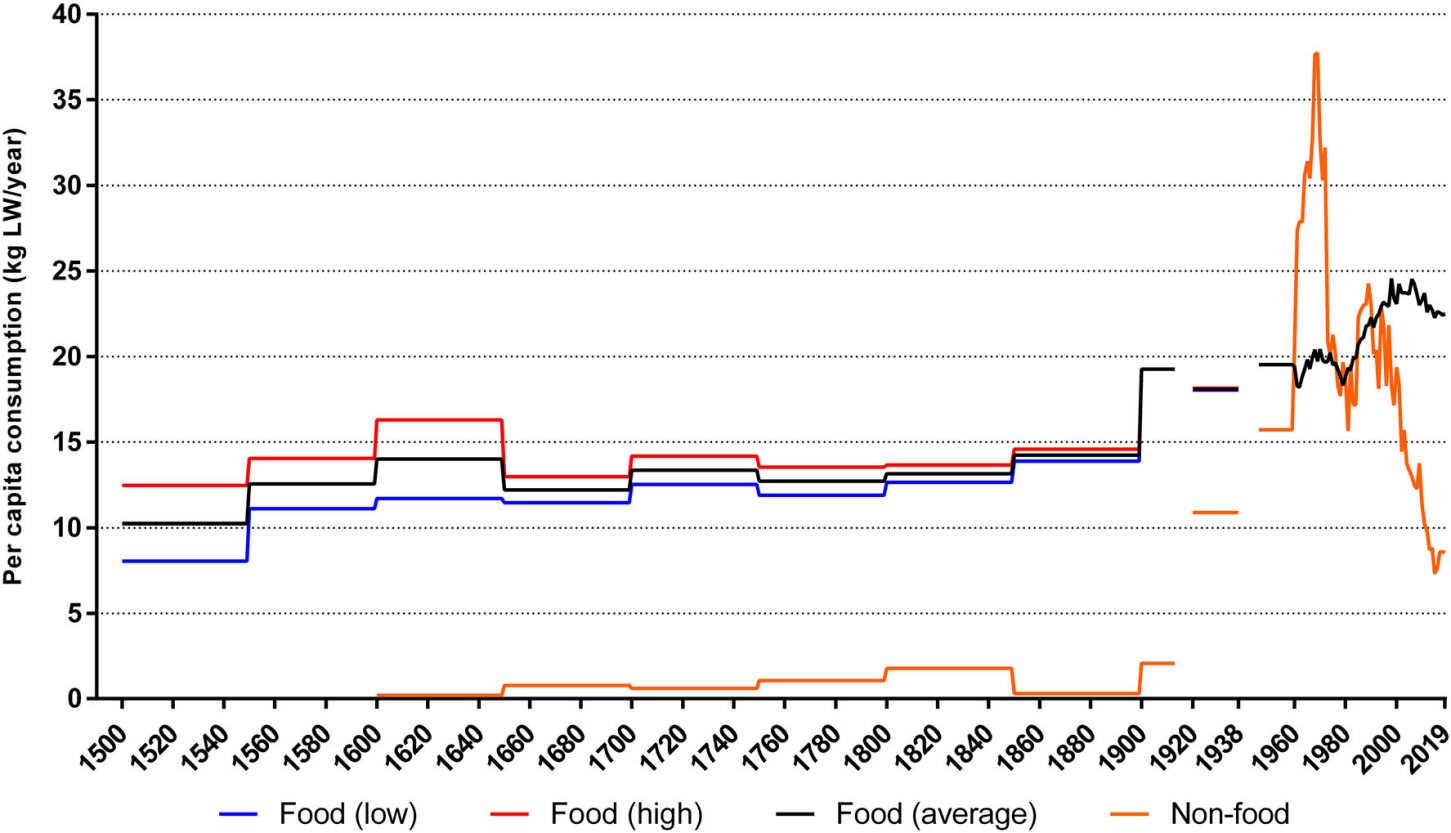
Estimated per capita food and non-food marine consumption, Atlantic Europe, 1500–2019 (kg LW per year). Upper and lower estimates of seafood consumption are given until 1900. Figures are weighted by the total population of each nation

On a per capita basis, Atlantic European consumption of marine life for food and non-food was relatively stable between 10 and 15 kg per person/year through the period 1500–1899 (Fig. 6). Per capita consumption accelerated from 1900 and peaked at 57 kg in 1968. The increase was predominantly due to a dramatic growth of non-food for agricultural and industrial uses, which accounted for 37 kg of total consumption. Since 1961, seafood consumption grew slightly from 18 kg to a peak of 24 kg in 1998. From 2017 to 2019, consumption remained around 31 kg, with non-food uses accounting for about 8 kg of this.

### The European marine footprint

Table 4 summarises the human marine footprint in Atlantic Europe through the last five centuries. Through the early modern age (1500–1800), the North Atlantic provided almost all the supplies for Europe. In the twentieth century, increasing demand and rising population figures caused Atlantic Europe to become increasingly dependent on food and whale oil imports leading to an expanded footprint on the world’s oceans. From 1980 onwards, we are not able to disaggregate North Atlantic and global provisions as declining Atlantic resources for human seafood consumption were compensated by global imports rather than direct fisheries landings. In the UK, landings and aquaculture equalled 89% of home consumption in 1975 but only 40% by 2019 (Harrison et al. 2023). In 2010, the European Union (EU28, including the UK) had a self-sufficiency rate of 45%, and for groundfish, including cod, the rate was 18% (EUMOFA 2014).

**Table 4:**
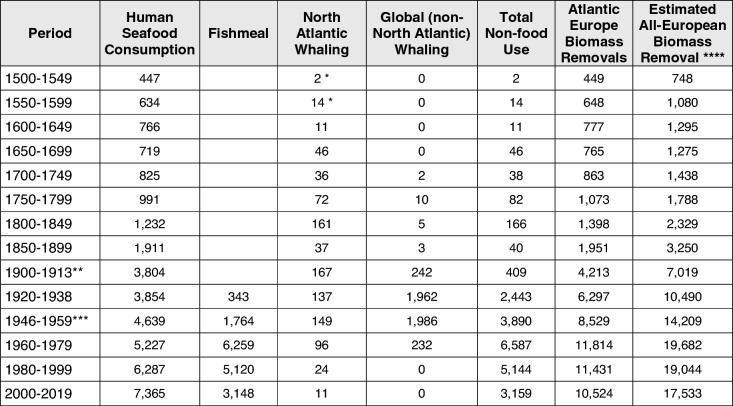
Estimated Atlantic European and All European marine footprint (excluding Russia), 1500–2019 (‘000 t LW)

Figures from 1961 onwards are from FAO Food Balance Sheets. See Fishmeal section in Supporting Doc for full figures

*North Atlantic Whaling figures for 1500–1549 and 1550–1599 incorporate values for early Basque whaling

**Excludes period 1914–1919 (First World War)

***Excludes period 1939–1945 (Second World War)

****The estimate of all-European Biomass removal is calculated as described in “Materials and methods” section

Over five centuries, the Atlantic European consumption of marine resources for food and non-food purposes increased by a factor of 40 to peak in the 1960s. Since then, marine consumption stagnated as non-food consumption declined, first as whaling was suspended and later by a decline of reduction of fish for meal and oil. Cumulatively, the Atlantic European consumption through five centuries amounted to 950 million t seafood and 388 million t non-food, a total of 1338 million t biomass.

All-European marine footprint estimates (Table 4) surpassed 1 million t by 1600. Consumption reached 7 million t by 1900 and peaked in 1960–1979 at almost 20 million t (seafood and non-food combined). Between 1500 and 2019, we estimate that all-Europe (excluding Russia) consumed at least 2250 million t of marine biomass, or probably well over 2500 million t considering the absence of data for non-food for many countries.

## Discussion

The human marine footprint of Atlantic Europe was significant well before being documented by modern consumption statistics. Long-term data reveal the enormity of pre-1960 marine removals previously not recognised. Specific cultural and industrial preferences impacted certain species by the nineteenth century. Europeans developed a preference for cod and herring for their own consumption, while North Atlantic cetaceans were targeted for lamp oil and grease. Most other fish species were less heavily exploited before the twentieth century. The expansion of human consumption between 1900 and 1913 was the most rapid through the last five centuries and signified profound changes to human uses of the ocean. Seafood consumption per capita almost doubled between 1899 and 1998. Non-food uses of whales and fish increased to account for half of human consumption—indirectly metabolised as oil for margarine and meal as feed for poultry and pigs. In recent decades, European marine consumption has declined despite a rapid growth of the global fish trade. While this paper makes progress towards a full accounting of consumption, there are still major unknowns, such as the harvesting of kelp and shellfish through time, and traditional, local and non-commercial fishing for which there is a lacuna of statistical evidence.

Knowing the marine footprint enables discussion of consumption drivers and ecological, socio-economic, and cultural impacts. Before 1850, total seafood consumption volumes largely mirrored demographic developments. Consumption of cod and herring, and possibly general levels of consumption, rose above population increases in the periods 1551–1620 and 1751–1800. Seafood consumption accelerated above population growth from 1880–1910 and 1950–1980. In recent decades, consumption declined relative to population. Demography is one of several drivers of total consumption. High-level figures conceal important regional, cultural, and social differences that require more detailed research into cultural foodways and how seafood production responded to factors like climate change and markets.

In the last 500 years, Atlantic Europe extended its marine footprint globally. This was part of the unequal shifting of biomass between continents that occurred during the so-called Columbian Exchange (Crosby 1986). We suggest that ‘ecological globalisation’, highlighting the impact of human mobility and exploitation, are apt concepts for characterising European marine exploitation of the North Atlantic and beyond (Barrett et al. 2020). As the authors of this piece, we therefore raise some questions to help identify areas for future inquiry. These questions bear directly on the wider global research agenda proposed by Holm et al. (2022).

“When were European home waters significantly fished down?” Bolster (2013) suggests this occurred by 1500 and resulted in Europeans seeking new fishing grounds in American waters. By our estimate, Atlantic European consumption around 1500 was about half a million tonnes of biomass. While a footprint of this order was relatively insignificant compared with twentieth-century consumption of 20 million tonnes, it is possible that specific inshore stocks were overfished. Early modern fishers were restricted to fish species and populations that were accessible to them with limited technology. Examples supporting this hypothesis include: the Spanish and French hake fisheries in the Celtic Sea being much reduced by the end of the sixteenth century (Hayes 2023), and the Atlantic grey whale becoming extinct about this time (NTNU University Museum 2022). However, commercial cod and herring catches in the North Sea remained steady and actually increased (Holm et al. 2022). It is unlikely that the ecological effect explains the scale of European migration of tens of thousands of fishers across the Atlantic for Newfoundland and the Gulf of Maine. This seems to have been a case of pull rather than push. Europeans were attracted to new resources rather than being pushed out by depleting home waters (Holm et al. 2019). Only future data gathering and modelling will answer the question.

“Was the rapid increase in consumption to 4 million t around 1900 of ecological consequence?” Most likely food webs and marine life cycles have been influenced by human exploitation. Many species have necessarily had their habitats and reproduction significantly transformed. The mechanisation of fishing technology, especially from the 1880s, and North Atlantic whale hunting in the early twentieth century may have caused ecological regime shifts that remained undetected because of the lack of long-term time series of ocean observation. These hypotheses are fertile testing grounds for future collaboration between historians, ecologists, and modellers.

“Is there a correlation between changes in climate and ocean conditions and seafood provision?” The only period during the last 500 years that saw a general decline in the European marine footprint was between 1650 and 1700, an exceptionally cold and stormy period (Clarke and Rendell 2009). Questions for future research include: “What was the likely impact of climate change on fishing effort and primary marine productivity?” And, “How did declining provisions impact human demography and societal resilience?” Thanks to the vastly expanded evidence base for 25 fisheries of the North Atlantic (Holm et al. 2021), the consumption data of the current paper, and multi-centennial price databases (Allen and Unger 2019), there is now an empirical basis for modelling and testing climate/environment/society.

“Did food preferences, a distinctive mark of cultural identity, drive human exploitation of the seas?” The Protestant Reformation ca. 1521–1560 is often flagged as a major deterrent of seafood consumption in early modern Europe as the practices of Lent and fish days were phased out (e.g., Fagan 2006). Our national-level analysis is not ideal for detecting small differences between religious groups, and the data do not indicate a clear difference between Protestant and Catholic countries. However, the marine footprint showed clear distinctions between northern and southern Atlantic Europe, with the northern countries experiencing a decline, while French, Spanish, and Portuguese consumption rose decisively. This apparently obvious verification of the impact of religion is negated by the experience of other countries. Ireland is the most extreme example of a Catholic country abandoning marine foods in the sixteenth century. Other cultural factors may be as important. French lightly salted “green” cod “came to represent the essence of the New World” for which customers were willing to pay a premium (Turgeon 2009). The contrast to English perceptions of saltfish is striking. Seafood for the masses were very cheap ‘Poor Johns’ (poor-quality salt cod) and dried herring (Thirsk 2006; Hayes 2023). Europe-wide, attitudes to fatty foods have apparently changed, driven by cultural norms. Seventeenth-century French cookbooks considered many traditional elite foods, like seabirds and sea mammals, inedible (Barlösius 2000). New ‘civilising’ tastes inspired European dining everywhere through the next century, narrowing the range of marine foods. The decline of marine consumption was particularly pronounced in England. In the sixteenth century, English food regularly included a diversity of shellfish as well as anchovies, eels, salmon, cod, and herring, even sturgeon for those who could afford it (Woolgar 2018). By the seventeenth century, such marine foods had largely disappeared from all but coastal and elite households. A narrow focus on a few commercial species, which developed in the last two centuries, most prevalently with the breakthrough of fish and chips shops for the urban working class, was a dominant legacy for the modern seafood market (Walton 1989). Only in recent decades have other species regained a place among seafoods.

Finally, “How did marine extractions contribute to European food security and demographic growth?” Food security increased in an egalitarian distribution system in periods of accelerated landings relative to demographic growth. However, the averages conceal huge national, regional, and socio-economic differences. Future research, including the ongoing 4-Oceans project (ERC Synergy 4-Oceans 2021), may reveal how the European and global marine footprint sustained human societies and answer the many other questions raised here.

## Conclusion

The history of the human marine footprint provides insights into the human impact on marine life and the significance of marine life for human societies. This study quantifies human consumption of seafood and non-food through the last five centuries and, by implication, total extractions of marine life from the North Atlantic. Crucially, we now know that although seafood consumption accelerated around 1900, levels of extractions were already substantial for several centuries before. The inference can be drawn that major impacts on marine food webs occurred well before the beginning of modern fisheries statistics in the early twentieth century. Global whaling and non-food fisheries contributed to a large and sustained European marine footprint through the twentieth century. A major future challenge will be quantifying the history of the global human marine footprint.
